# Flexural Performance of Sisal Fiber Reinforced Foamed Concrete under Static and Fatigue Loading

**DOI:** 10.3390/ma13143098

**Published:** 2020-07-10

**Authors:** Jun Huang, Guoxin Tian, Peiyan Huang, Zhanbiao Chen

**Affiliations:** 1 College of Civil Engineering and Architecture, Wenzhou University, Wenzhou 325035, China; junhd@wzu.edu.cn; 2 School of Civil Engineering and Architecture, Guangxi University of Science and Technology, Liuzhou 545006, China; tgx_0531@163.com; 3 School of Civil Engineering and Transportation, South China University of Technology, Guangzhou 510640, China; chenzb@scut.edu.cn

**Keywords:** sisal fiber, flexural performance, fatigue life, foam concrete

## Abstract

To improve the weak mechanical properties of the foamed concrete that resulted from a large number of pores, a plant fiber is used as fill with the matrix. In this study, five contents of sisal fiber are added into the foamed concrete to investigate the static and fatigue performance of composites. The static and fatigue experimental results show that the sisal fiber can improve the mechanical properties of the foamed concrete. When the content of sisal fiber is less than 0.15%, the higher the content of sisal fiber, the greater the bending strength and fatigue life of the foamed concrete. In contrast, if the sisal fiber content is larger than 0.15%, the bending strength and fatigue life decrease with the increasing fiber content. From the regression analyses, the results show that the double linear relationships exist not only between the sisal fiber content and the flexural strength, but also between the sisal fiber content and the fatigue life of the foamed concrete. In this study, the optimal content of sisal fiber mixed in the foamed concrete can be inferred to be 0.133%.

## 1. Introduction

For the excellent physical properties, the foamed concrete is extensively used in civil engineering. In 1923, a swede Axel Eriksson achieved the first patent related to the foamed concrete [1]. After that, the foamed concrete research had been greatly developed. In 1996, the work of the foamed concrete in bridge construction is conducted by Pickford C. et al. [2]. In 1998, the light weight foamed concrete was investigated by Byun K.J. et al. [3] when polymer foam agent was used. In recent years, although the applications of the foamed concrete are still conducted, such as the low density foamed concrete used in filling engineering, the high density foamed concrete is used in insulated structures (Ramamurthy K. [4]). More and more studies are focused on the mechanical properties of the foam concrete.

Tikalsky P.J. et al. [5] developed a modified freeze–thaw test procedure based on ASTM (American Society for Testing and Materials) C666 to investigate the freeze–thaw durability of the foam cellular concrete. Nambiar E.K.K. et al. [6] presented that air-void can influence the mechanical properties of the foamed concrete, especially, the volume, the size, and the spacing of air voids. Air-void shape has no effects on the properties of the foamed concrete. Thereafter, Just A. et al. [7] used petrographic microscopy and a scanning electron microscopy to study the influences of aluminum powder and chemical additives on the properties of the micro-structure of the foamed concrete. Another case, during the cement hydration, the heat generated in the foamed concrete is different from normal concrete. Tarasov A.S. et al. [8] suggested the heat evolution process of the foamed concrete can be controlled by optimizing the mix composition. Othuman M.A. et al. [9] adopted the experimental and analytical method to quantify the thermal properties of light weight foamed concrete at high temperatures. Using three types of alkali activators, ground granulated blast-furnace slag was selected to improve the mechanical properties of the foamed concrete, the results showed that the compressive strength of the alkali-activated slag foamed concrete is higher than that of the normal foamed concrete (Yang K.H. [10]). Based on the low density, the high strength-to-weight ratio, energy conservation, lower labor cost, etc., Amran Y.H.M. et al. [11] provided a review on the constituents, mechanical properties of the foamed concrete and a comprehensive insight in actual applications of the foamed concrete. 

Although the foamed concrete has many advantages, such as light weight, sound insulation, heat insulation, water proof, etc., its strength is very low. To improve mechanical properties of the foamed concrete, varied types of fibers are added into the matrix. Different fiber has different technical characteristics, however, the content and the type of the fiber will play an important role on improving the mechanical properties of the foamed concrete. Kyung-Ho L. et al. [12] investigated the improvements of four fiber contents from 0.3 kg/m^3^ and 0.6 kg/m^3^ to 1.2 kg/m^3^, it showed that the optimum content of fibers is 0.6 kg/mm^3^. When the content reaches 0.9 kg/m^3^ or more, the agglomeration of fibers will occur to decrease the mechanical properties. Further study was presented by Falliano D. et al. [13] on the compressive strength and the flexural strength of the foamed concrete with three contents of fibers, when the content of fibers reaches 2% and 5%, the flexural strength of the foamed concrete can increase 13% and 70%. On the other hand, the varied fiber has different mechanical properties. If the content of fiber is constant in volume fraction (*V_f_*) 2%, the flexural strength of composites will increase by 327%, 382%, 430%, and 485% for the polypropylene fibers, bare steel fibers, zinc-coated fibers, and brass-coated fibers (Corinaldesi V. et al. [14]). Mastali M. et al. [15] compared the effects of polyvinyl alcohol fibers, polypropylene fibers, and basalt fibers on the mechanical properties of composites. It showed that the polyvinyl alcohol fiber was more significant than that of other fibers, as the polyvinyl alcohol fiber has an efficient bonding with the matrix. In addition, Flores-Johnson E.A. et al. [16] used uniaxial tensile test to measure the tensile mechanical properties of polyvinyl alcohol fiber reinforced foamed concrete. It showed that the fiber drastically enhanced the tensile modulus, strength, and yield strain of the foamed concrete and also avoids a brittle failure of the composites. For the polypropylene microfiber, it is proved by Mamun M. et al. [17] that it can lead to better post-peak response even when exposed to a sulfate environment. Keerio M.A. et al. [18] selected plastic fiber with the content of 0.6% to investigate the ultimate load and load deflection profiles of composites, it showed that the fiber reinforced foamed concrete had higher ultimate load than that of neat foamed concrete. Comparing single type of fibers, hybrid modes have more advantages to improve the mechanical properties of the foamed concrete. Devid F. et al. [19] adopted bi-directional glass-fiber grids to strengthen the tensile zone of the beam and short polymer fibers to embedded in the matrix with the volume fraction of 2% and 5%. For all cases, the flexural capacity of the foamed concrete is efficiently improved. Dawood E.T. et al. [20] presented to mix with the glass fiber and the polypropylene fiber to improve the mechanical properties of the foamed concrete. The hybrid percentages of two fibers are taken as 0.2% + 0.6%, 0.4% + 0.6%, 0.2% + 0.1%, and 0.4% + 1%. The results proved the glass fiber is more efficient than that of the polypropylene on the compressive strength and the flexural strength. However, the latter improve the flexural toughness of the foamed concrete better than the former. Thereafter, Dawood E.T. et al. [21] further demonstrated the effects of hybrid carbon fibers and polypropylene fibers on improving the mechanical properties of the foamed concrete under different temperature. In general, with the temperature increasing, the mechanical properties will decrease, compared with the carbon fiber reinforce foamed concrete, the polypropylene reinforced foamed concrete is more sensitive to the elevated temperature. Although many types of fibers have been used to enhance the strength of the foamed concrete, the research on the sisal fiber reinforced foamed concrete is very lack. Despite this, in terms of a high resolution image capturing procedure and the bridging function of sisal fibers, Silva, et al. [22] studied the crack spacing of sisal fiber reinforced cement composites under tensile and bending responses. After that, the authors further researched the pull-out behavior of sisal fiber from a cement matrix, and investigated the tensile fatigue behavior of long aligned sisal fiber reinforced cement composites, the results showed that the sisal fibers can arrest and bridge the cracks even when the composite was subjected to 106 cycles at 50% of ultimate tensile strength [23,24].

The sisal fiber comes from the plant, with high environmental protection, easy degradation, low cost, adding it to the foamed concrete matrix to improve its flexural and fatigue performance, has important scientific significance and broad application prospects. In this study, the natural sisal fiber is used to improve mechanical properties of the foamed concrete. The task of the research is to find the effects of the sisal fiber on the static and fatigue performance of the foamed concrete. It includes three steps, the first is the static bending tests and then compressive tests will be carried out, from which the bending strength and the compressive strength can be gotten. The second, the fatigue performance tests of the concrete with five sisal fiber contents and the neat concrete are completed. Finally, the relationships between the fatigue life and the strain of, the fatigue equations of the composite will be conducted.

## 2. Test Preparation

To study the effects of the sisal fiber on mechanical properties of composites, the bending tests are divided into two parts. The first part is the static bending test and the second part is the bending fatigue test. The static bending tests include six groups, and each group has three specimens used to get the bending strength, from which the stress/loading levels of the fatigue tests can be determined. The first group of the static tests is used as reference group which has no any sisal fiber. In this group, the specimens are denoted as 0-1, 0-2, and 0-3. Except the first group, the other five groups contain five sisal fiber content of concretes, and the corresponding sisal fiber volume ratios are applied as 0.05%, 0.1%, 0.15%, 0.2%, and 0.3%. The numbers of all the static test specimens are listed in Table 1.

Similarly, the fatigue tests also include six groups, and each group chooses nine specimens to complete fatigue tests under three stress/loading levels of 0.75, 0.80, and 0.90. The numbers of the fatigue specimens are listed in Table 1 too. For all the specimens, the three-point bending beam will be selected, and the size is taken as 100 mm × 100 mm × 300 mm. The load is applied at the midpoint of the beam, and each support point is left away from the end of the beam by about 50 mm.

In this study, the ordinary Portland cement labeled 42.5 (Guangxi Yufeng group, Liuzhou, China) is selected as the binding material. To improve the compactness of the concrete and save the cement, fly ash and the silica fume are also used. Silica fume has a smaller fineness, and the average particle size ranged from 0.1 to 0.3 microns, which acts as a good filling enhancement effect. The standard sand is selected as aggregate, and the water-binder ratio is taken as 0.45. To get the foamed concrete, hydrogen peroxide foaming agent (30 L) and calcium stearate foam stabilizer (2 kg) are used in tests, at the same time, water reducing agent is selected to increase the strength of the composites. The mix ratio of the neat foamed concrete is listed in Table 2. Changing the sisal fiber content, the mix ratios of material components for other groups can be gotten. The sisal fibers with 5 volume fractions will be used in these tests, the mechanical properties of the sisal fiber are listed in Table 3. Here, the sisal fiber must be cut to the short length of 10 mm when they are added into the matrix.

## 3. Static Tests

### 3.1. Test Procedure

Making the foam concrete includes the preparation of the mortar, configuration of the foam agent, mixing with sisal fiber and stirring and vibrating. In detail, firstly, the cement, standard sand, fly ash, and the silica fume are poured into the mortar mixer to stir for 1.5 min, and then, the different volume fraction sisal fibers are added into the matrix to stir for 2 min. After that, other materials, such as the hydrogen peroxide foaming agent are mixed in the compound slowly. All the materials are stirred for 3 min again to ensure they are well mixed.

The static bending tests are carried out on the hydraulic universal testing machine WEW-300B (Jinan testing machine factory, Jinan, China) with the computer controlling. The distance of two support points is 200 mm, the strain gauges glued in the specimen are connected with the static resistance strain gauge. At the mid-span, three strain gauges are glued on the side of the beam to measure the strains of the upper, lower, and the neutral layer. The positions at the upper and the lower are close to the top and the bottom about 15 mm. At the same time, two strain gauges are glued at the positions close to the support points. The distances between the strain gauge and the support point or the bottom are also 15 mm. The force loading is applied as the loading mode and the loading rate is 0.02 kN/s.

### 3.2. Bending Strength of Foam Concrete

To conveniently compare the bending strength of the foamed concrete with different sisal fiber contents, all the testing results are listed in Table 4. Here, each strength or strain is the average value from the three specimens.

**Figure 1:**
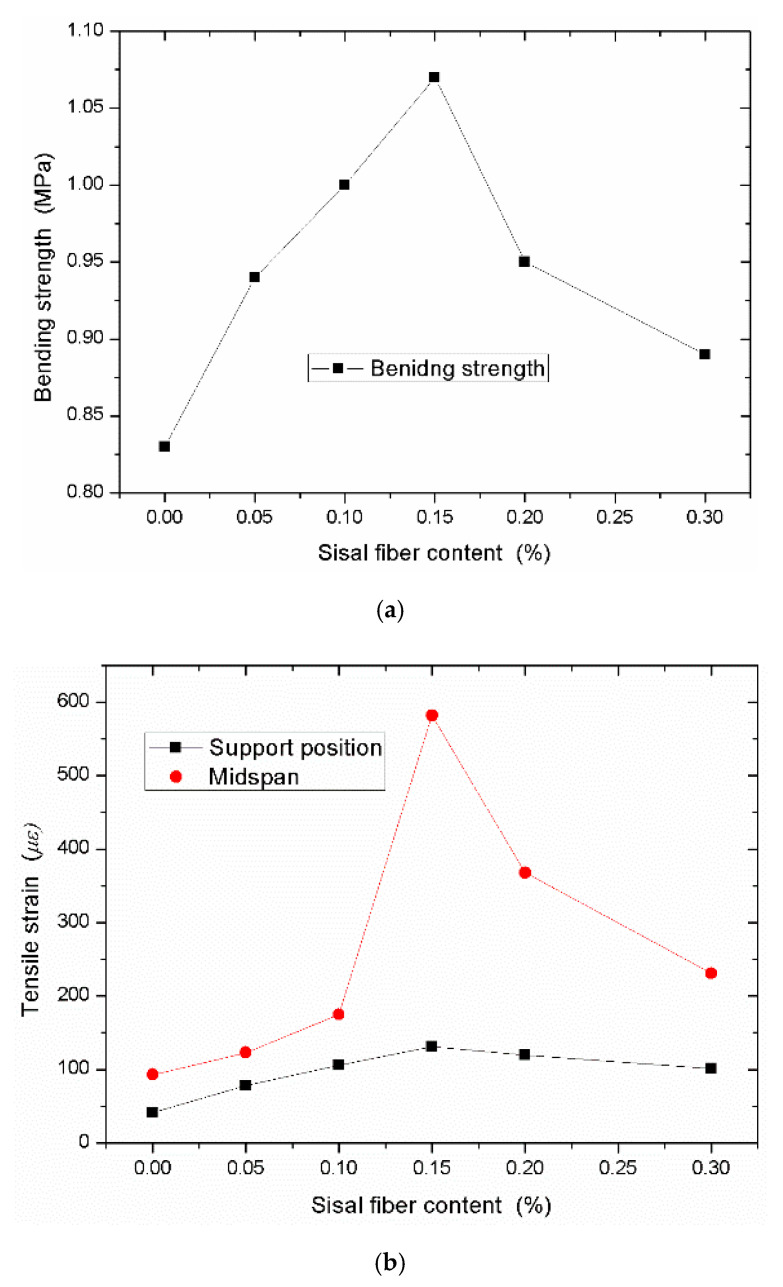
Bending strength of the foamed concrete with different sisal fiber contents: (**a**) The bending strength and (**b**) the ultimate strains at the specified positions.

From the above testing data, it can be seen that the bending strength of composites added with different sisal fibers are higher than that of neat foamed concrete. On the other hand, because of the toughness of the sisal fiber, the ultimate strains of the sisal fiber reinforced the foamed concrete at the specified positions are also larger than that of the neat foamed concrete. The specified positions glued the strain gauges are located at the midspan of the beam and near the support point. If all the responding data with different sisal fiber contents are plotted in the diagrams, it is more convenient to compare the improvement of the sisal fiber on the bending performance of composites (Figure 1). 

**Figure 2:**
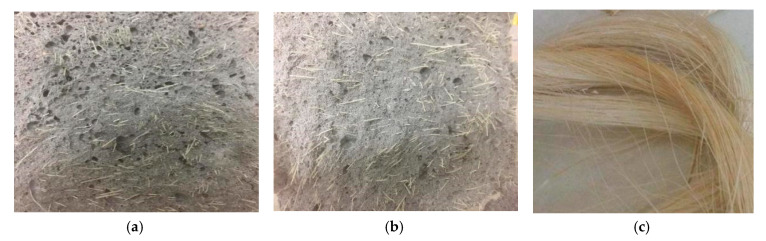
Failed sections of sisal fiber reinforced foam concrete. (**a**) Sisal fiber content of 0.2%. (**b**) Sisal fiber content of 0.3%. (**c**) Original sisal fibers.

Figure 1a shows that the bending strength of composites increases at the beginning, and then decreases with the increasing sisal fiber content. Comparing the foam concrete, the sisal fiber has higher Young’s modulus and fracture strength, so the sisal fiber can improve the mechanical properties of composites. However, when the sisal fibers are mixed into the matrix, they are not easy to disperse and entangle together which decreases the improvement effects of the sisal fiber on the bending strength. On the other hand, the sisal fiber can absorb water to produce the volume expansion, which decreases the bending strength of composites. Figure 1b has similar results. The tensile strain of the specimen reaches the maximum value when the volume fraction of the sisal fiber is taken as 0.15%. This means the sisal fiber can improve the toughness of the composites if the fibers can be dispersed in the matrix homogeneously. From Figure 1b, the tensile strain at the mid-span is larger than that of the support position. Compared with the tensile strain of the neat foamed concrete, at the mid-span position, the tensile strains of the specimens with the volume fraction of 0.05%, 0.1%, 0.15%, 0.2%, and 0.3% are 1.32, 2.42, 6.26, 3.96, and 2.48 times that of the tensile strain of the neat specimen. During the breaking of the specimen, sisal fibers can play a bridging role, which lets the composites have a ductile fracture. For all the static bending tests, the initial crack happened at the bottom of the beam. If the first crack occurs, the crack will run through the whole cross section. The neat foamed concrete will make a crisp sound when it breaks. With the volume fraction of the sisal fiber increasing, the breaking sound of the specimen will gradually decrease. Even the specimen does not break when the crack is enlarged. It is especially obvious for the volume fraction of the sisal fiber of 0.3%. From the failure sections (Figure 2) of the specimens with high sisal fiber content, the fibers that are embedded in the matrix can be seen. When the beam begins to crack and fracture, the sisal fiber will be pulled out from the matrix. During this process, the sisal fiber can hinder the crack propagation and it will consume more energy if the specimen fractures. However, high content sisal fibers are easy to agglomerate together, which decreases the effects of the improvement.

### 3.3. Effect of Sisal Fiber Content

**Figure 3:**
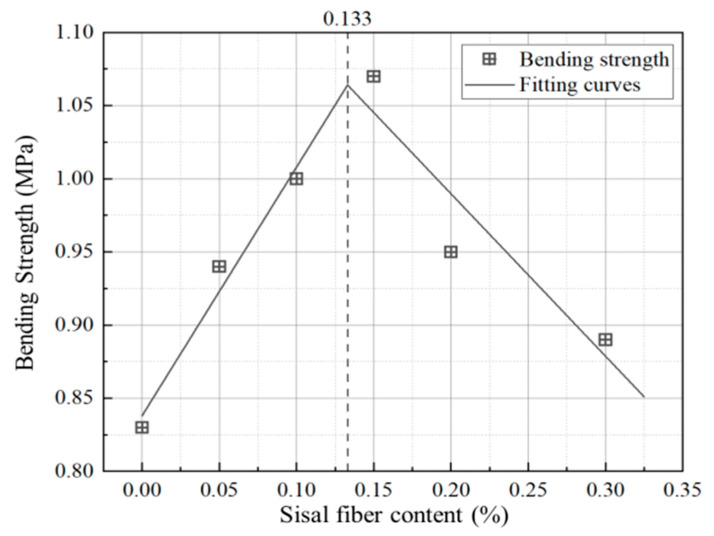
Relationship between bending strength and sisal fiber content.

In order to find the optimal sisal fiber volume fraction, the bi-linear regression on the bending strengths of the specimens is carried out. From the first three points, the ascent line is fitted and can be extended over the horizontal coordinate of 0.10. Similarly, in terms of the last three points, the second fitting line can be gotten. The two fitting curves (Figure 3) can produce the intersect point. It is easy to find the fitting peak with the maximum bending strength of 1.06 MPa and the corresponding sisal fiber content of 0.133%. If the intercepts and the slopes of the two fitting lines are considered, the fitting bending strength equation related to the sisal fiber content can be gotten, as shown in Equation (1).

(1) $$f_{t}=\{\begin{array}{c} 0.838+170C,\;\;\;\;C<0.00133\\ 1.06-111(C-0.00133),\;\;\;\;C\geq 0.00133 \end{array}$$

where, *f_t_* is the bending strength and the *C* is the content of the sisal fiber.

From the determination coefficients of the two fitting lines, the average value is obtained as 0.922, so the correlation coefficient can arrive at 0.960 which is close to 1.0. Thus, the optimal sisal fiber content obtained with this method is convincing.

## 4. Fatigue Performance of the Foamed Concrete

### 4.1. Fatigue Tests

In this study, the materials and specimens used in the fatigue experiments are the same as those used in the static bending tests. In terms of the above static bending tests, 18 specimens in six groups are used in the fatigue tests. The bending load *P*_u_ can be used to determine the stress levels for fatigue tests. Three stress/loading levels (*S*_R_ = *P*_max_/*P*_u_) of 0.75, 0.80, and 0.90 are selected, the loading forces are gotten from the bending strength (Table 5). To complete these tests, the loading frequency is taken as 10 Hz and the sinusoidal waveform is applied. Considering the dead loading effect of bridge structures, the stress ratio is taken as *R* = 0.5.

**Figure 4:**
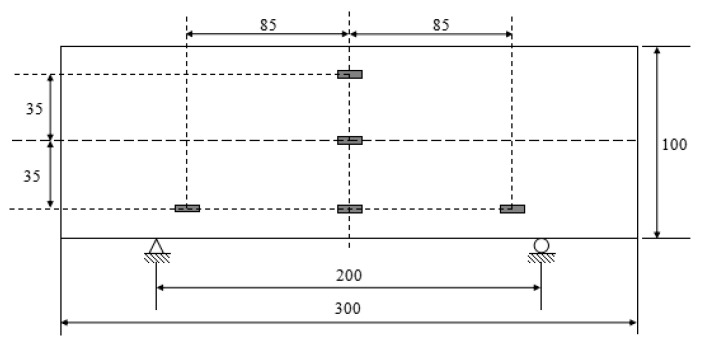
Locations where strain gauges are installed, units in mm.

In order to get the relationship between the strain and the fatigue performance of the specimens, five strain gauges are glued in the middle of the beam to measure the tensile strain and compressive strain, as shown in Figure 4. One strain gauge is glued in the neutral layer of the beam to check the deformation. Two strain gauges used to measure the tensile and compressive deformation are glued to be away the bottom and the top of the beam about 15 mm. The other two strain gauges are glued at the positions which are away the support points of 15 mm. The strains are acquired by the dynamic resistance strain gauge TMR7200 (Tokyo measuring instruments laboratory co., ltd., Tokyo, Japan), and the sampling frequency is 100 Hz. 

All fatigue tests are completed on the electro-hydraulic serve fatigue testing machine with the type of HYS-100 (Changchun Hao yuan testing machine co., ltd., Changchun, China). Experimental data such as maximum and minimum load, mid-span displacement of the specimen, loading cycle number (fatigue life) *N*, etc. are recorded automatically by the testing system.

### 4.2. Experimental Results and Analyses

**Figure 5:**
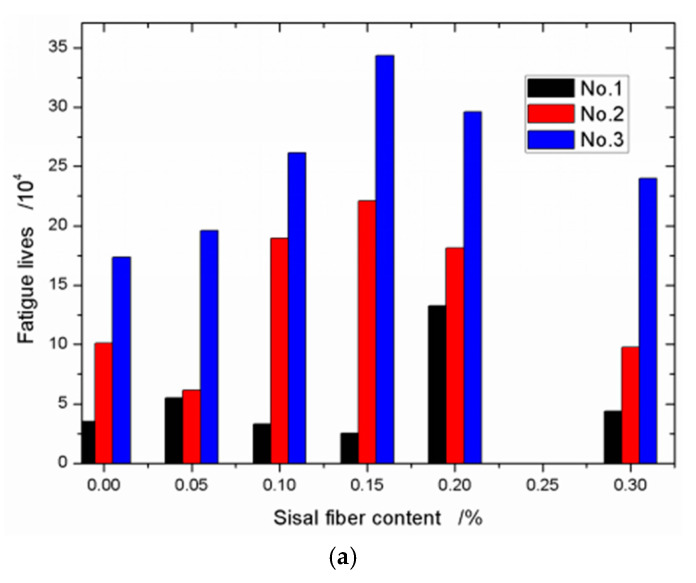

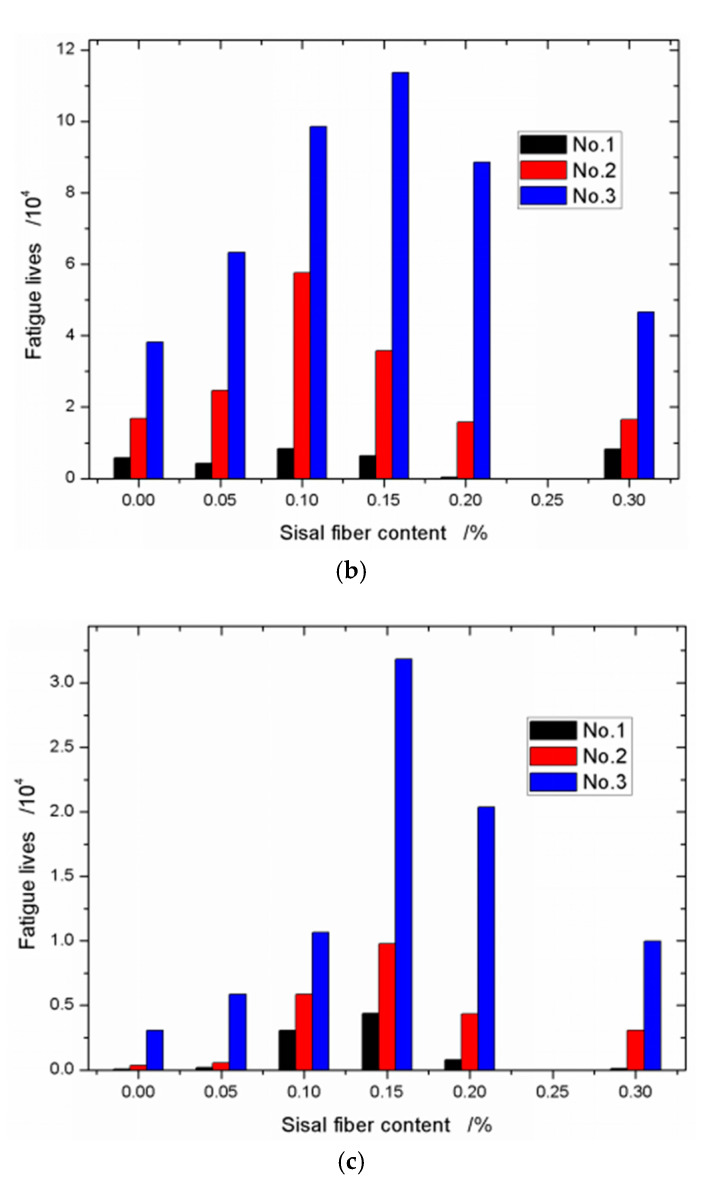
Fatigue lives with different sisal fiber contents (**a**) Stress level *S*_R_ = 0.75; (**b**) Stress level *S*_R_ = 0.80; and (**c**) stress level *S*_R_ = 0.90.

For each stress level and each sisal fiber content, there are three specimens are tested. In order to compare these test results conveniently, all the data are plotted in Figure 5 in the form of histogram. As shown in Figure 5, it is easy to see that the fatigue experimental data are very discrete. This case is caused by many factors, the first, materials are not homogeneous, and each specimen has different micro-defects, voids, etc. In fact, each specimen has different static bending strength, which will also affect the fatigue lives of the specimens. The second, during the test process, there are many indeterminate factors such as temperature, age, the accurate position of the specimen to affect the final results. In spite of this, the fatigue life of a specimen decreases with the stress level increasing, and in general, the sisal fiber can enhance the fatigue lives of composites. As an example of Figure 5a, the improvement of the sisal fiber on the fatigue life of composites tends to rise first and then fall. Comparing the static bending tests, the similar phenomenon is that the maximum fatigue life of composite occurs on the specimen with the sisal fiber content of 0.15%. This case can be used to explain Figure 5b,c too.

#### 4.2.1. Tensile Strain Curves

**Figure 6:**
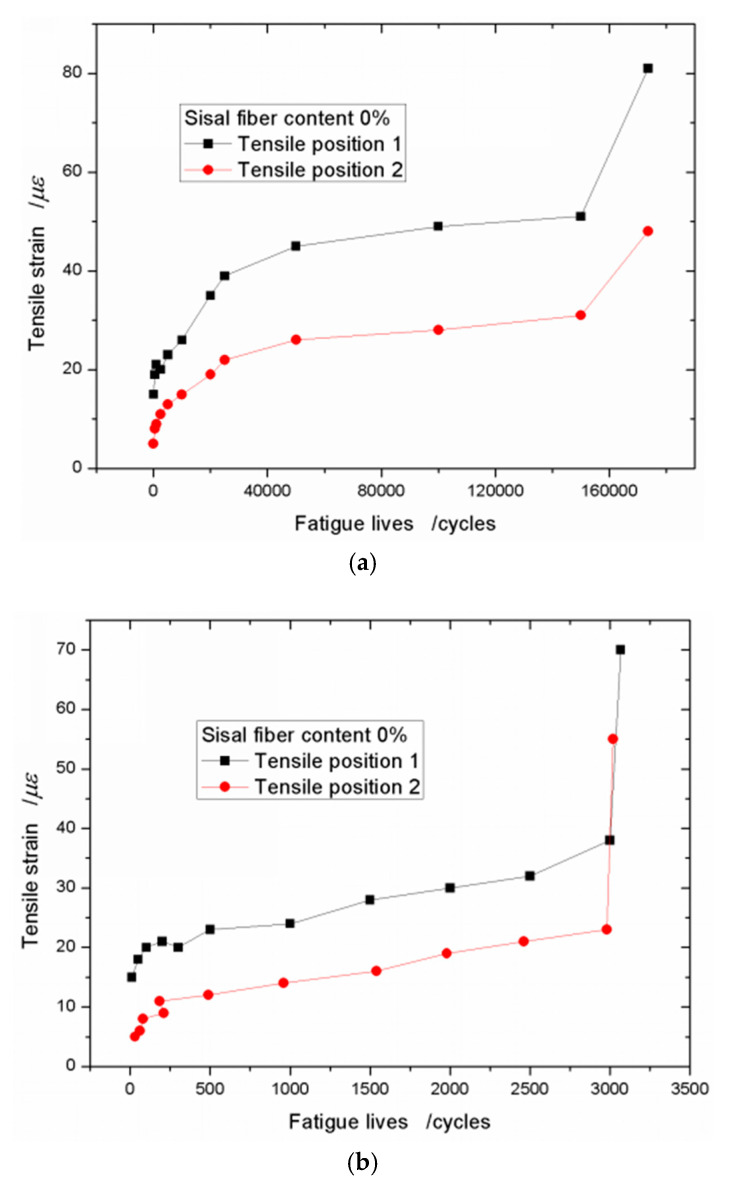
Tensile strain–fatigue life curves with sisal fiber content of 0% under different stress levels. (**a**) *S*_R_ = 0.75 and (**b**) *S*_R_ = 0.90.

In order to get the relationship between the strains and the fatigue lives of the specimens, the tensile strains at the middle of the beam (Tensile position 1) and the tensile strains at one side close to the support point (Tensile position 2) are as samples to be discussed. The tensile strain–fatigue life curves with no sisal fiber are plotted in Figure 6. From Figure 6a, whatever position 1 or position 2, the curves both show the three stages changing. The first stage, the strain increases very fast, and then, the curves tend to be stable and the strains grow slowly. At the third stage, the strain increases rapidly, finally, the specimen breaks. This breaking phenomenon of the foamed concrete is similar with that of the plain concrete. When the stress level is 0.90, the tensile strain–fatigue life curve is like Figure 6a, and the curves are plotted in Figure 6b.

**Figure 7:**
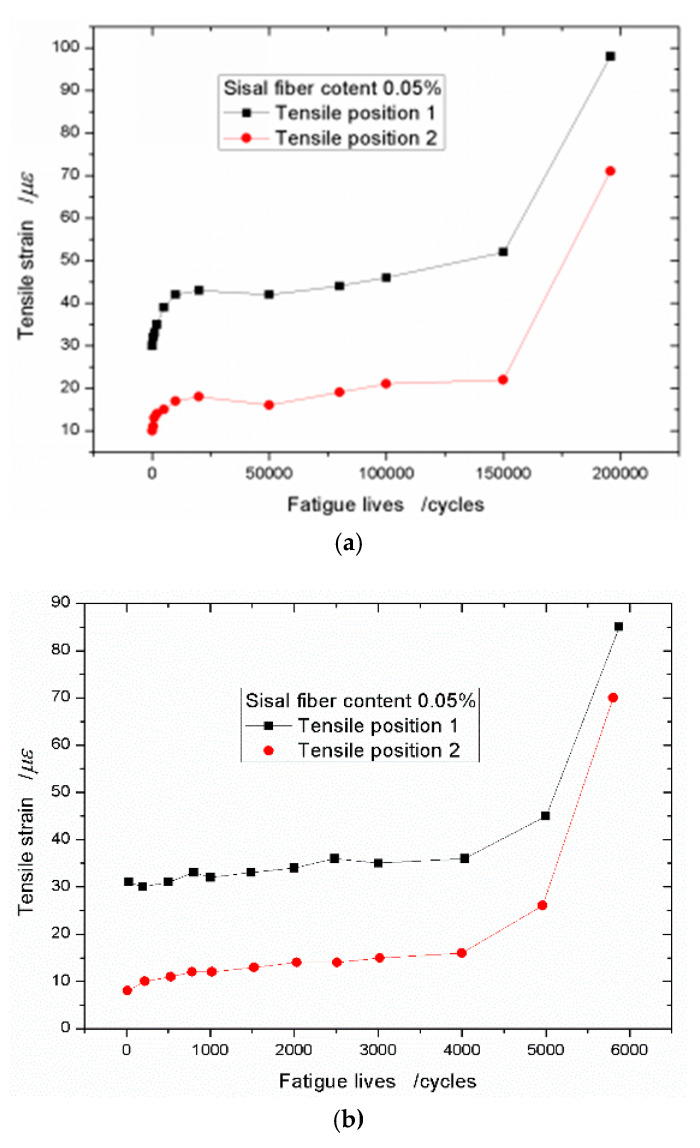
Tensile strain–fatigue life curves with sisal fiber content of 0.05% under different stress levels (**a**) *S*_R_ = 0.75 and (**b**): *S*_R_ = 0.90.

**Figure 8:**
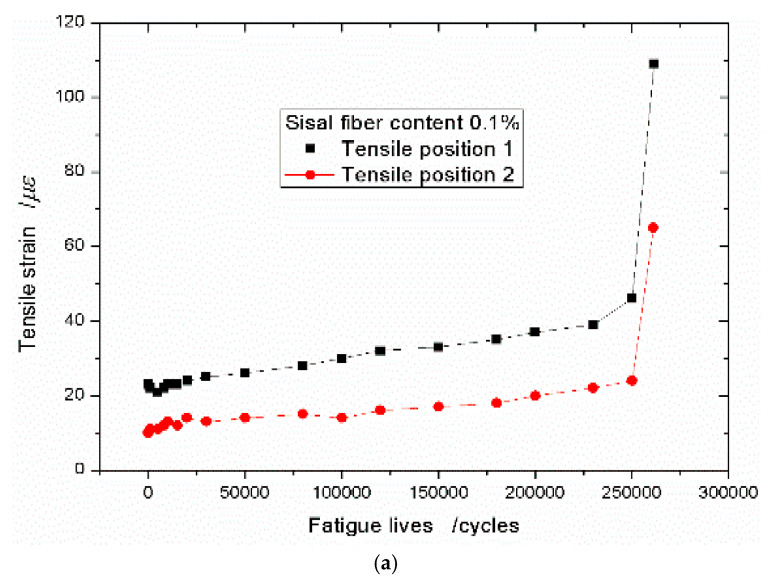

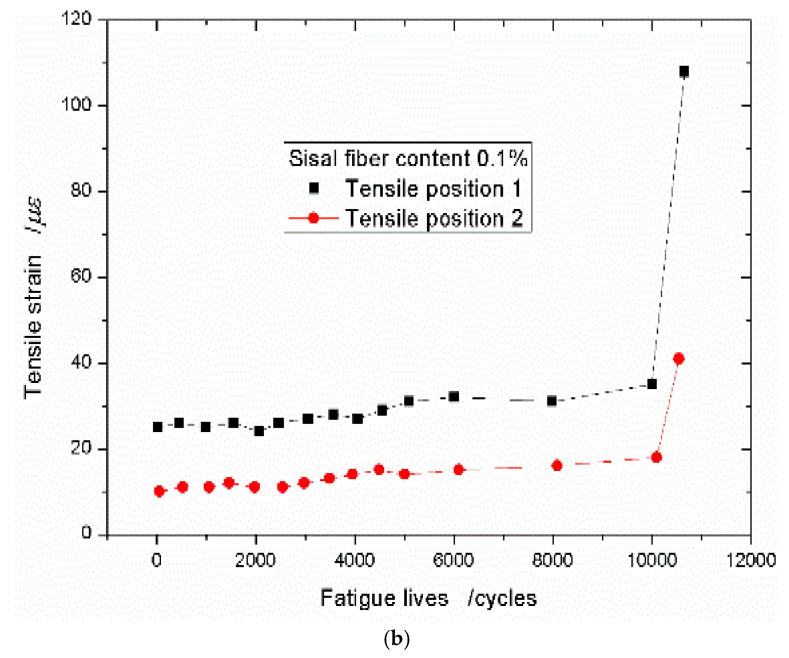
Tensile strain–fatigue life curves with sisal fiber content of 0.1% under different stress levels. (**a**) *S*_R_ = 0.75 and (**b**) *S*_R_ = 0.90.

When the content of the sisal fiber is taken as 0.05% and the stress level is 0.75, the strain data are plotted in Figure 7a. It shows that the strain increasing trend still appears the three stages, however, comparing Figure 6a, the first stage is shorter. As the tensile strain grows rapidly and slightly, and then it quickly enters the second stage of steady rise. It is different with Figure 7a, Figure 7b shows the tensile strain increasing trend with two stages. At the beginning, the tensile strain will enter the stage of steady rise directly. In this case, the stress level is 0.90, so it can be see that the sisal fiber has more significant effect on the tensile deformation of the foamed concrete when the stress level is increased. If the sisal fiber content continues to increase, no matter what value the stress level takes, 0.75, 0.80, or 0.90, the tensile strain increasing trend appears two stages. It further proves that the sisal fiber efficiently prevents the cracks early propagation. Figure 8 shows the tensile strain–fatigue life curves of the concrete with the sisal fiber volume fraction of 0.1%.

**Figure 9:**
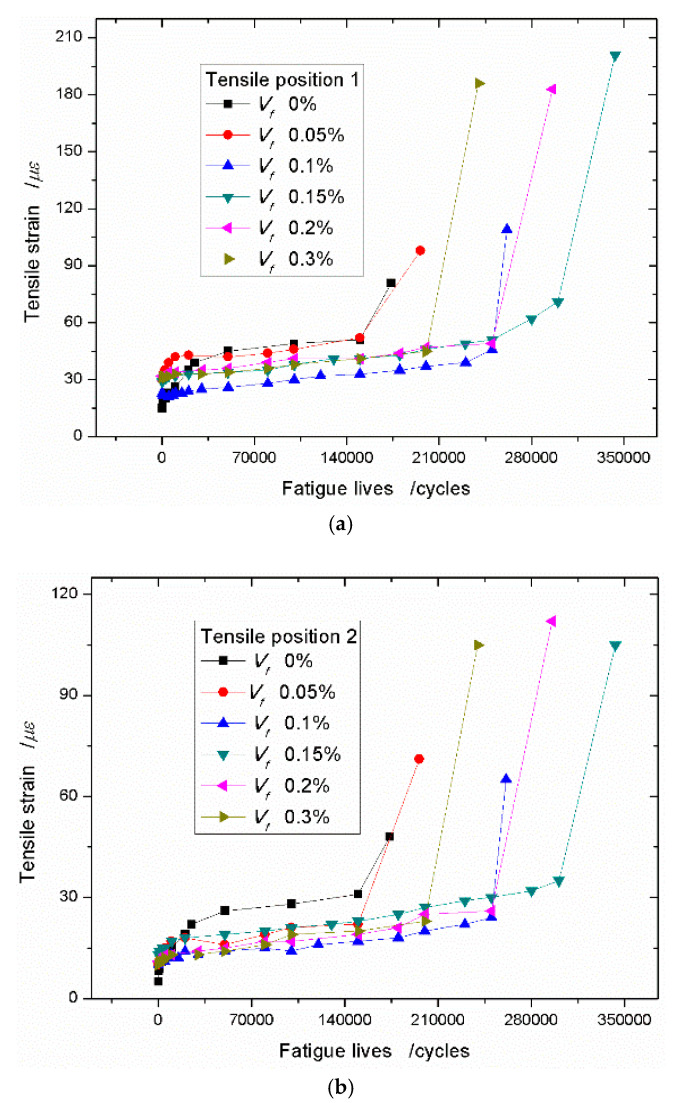
Tensile strain–fatigue life curves with different sisal fiber content under the stress level of 0.75. (**a**) Tensile position 1; (**b**) Tensile position 2.

Because the foamed concrete has many voids and micro-cracks, it has the low density and low strength. When the sisal fibers are added into the matrix, the sisal fiber can play a bridging role to resist the deformation of the composites if the cracks grow under the external force. This means that the sisal fiber can absorb more energy as the composites break. In order to compare the strain increasing trend and the effects of resisting crack propagation, the tensile strain–fatigue life curves with the different volume fraction of sisal fibers are placed into one figure. Under the stress level of 0.75, the tensile strain curves with varied sisal fiber content at the position 1 are plotted in Figure 8. Figure 9a shows the sisal fiber appears to have higher toughness and higher deformation than that of neat foamed concrete. At the same time, the sisal fiber can largely increase the fatigue life of composites, especially when the sisal fiber content is taken as 0.15%. When the sisal fiber content of 0.15% is selected, the fatigue life of the composite is 1.98 times that of the neat foamed concrete. On the other side, the ultimate tensile strain at the position 1 arrives at 201 *με* and far greater than the value 81 *με* of the neat foamed concrete. When the sisal fiber volume fraction continues to increase, the fatigue life of composite and the ultimate tensile strain at the position 1 decrease slightly. This case is caused by the agglomeration of sisal fibers. For position 2, close to the support point, the similar improvements of sisal fibers on the tensile strain and the fatigue lives of the composites are plotted in Figure 9b.

**Figure 10:**
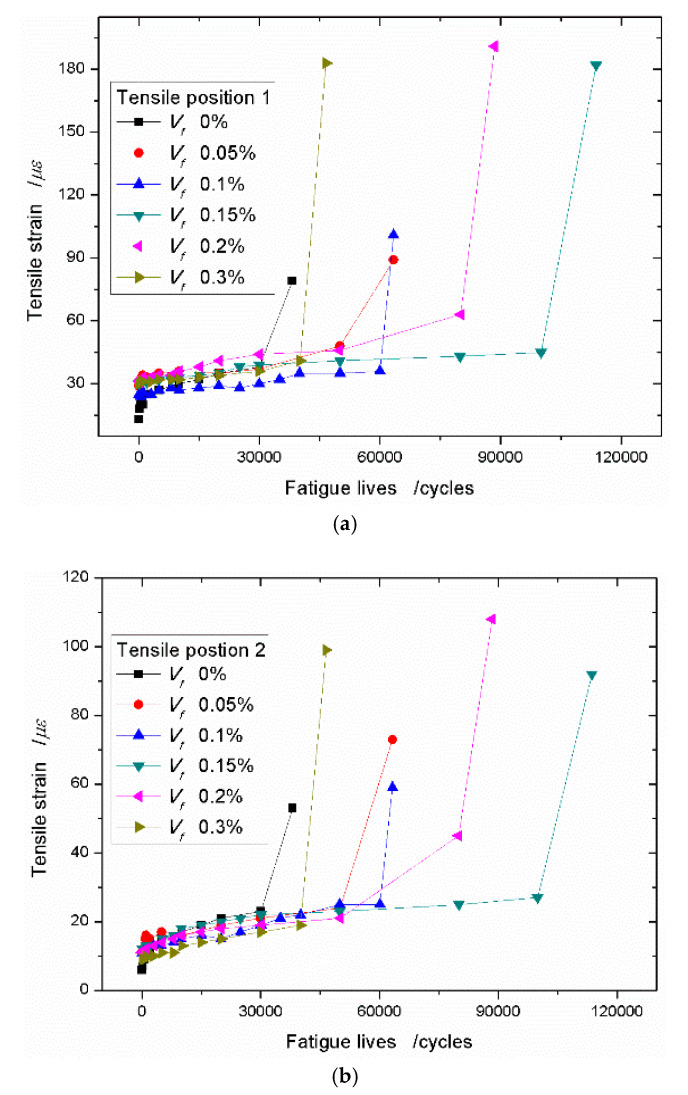
Tensile strain–fatigue life curves with different sisal fiber content under the stress level of 0.80. (**a**) Tensile position 1; (**b**) Tensile position 2.

**Figure 11:**
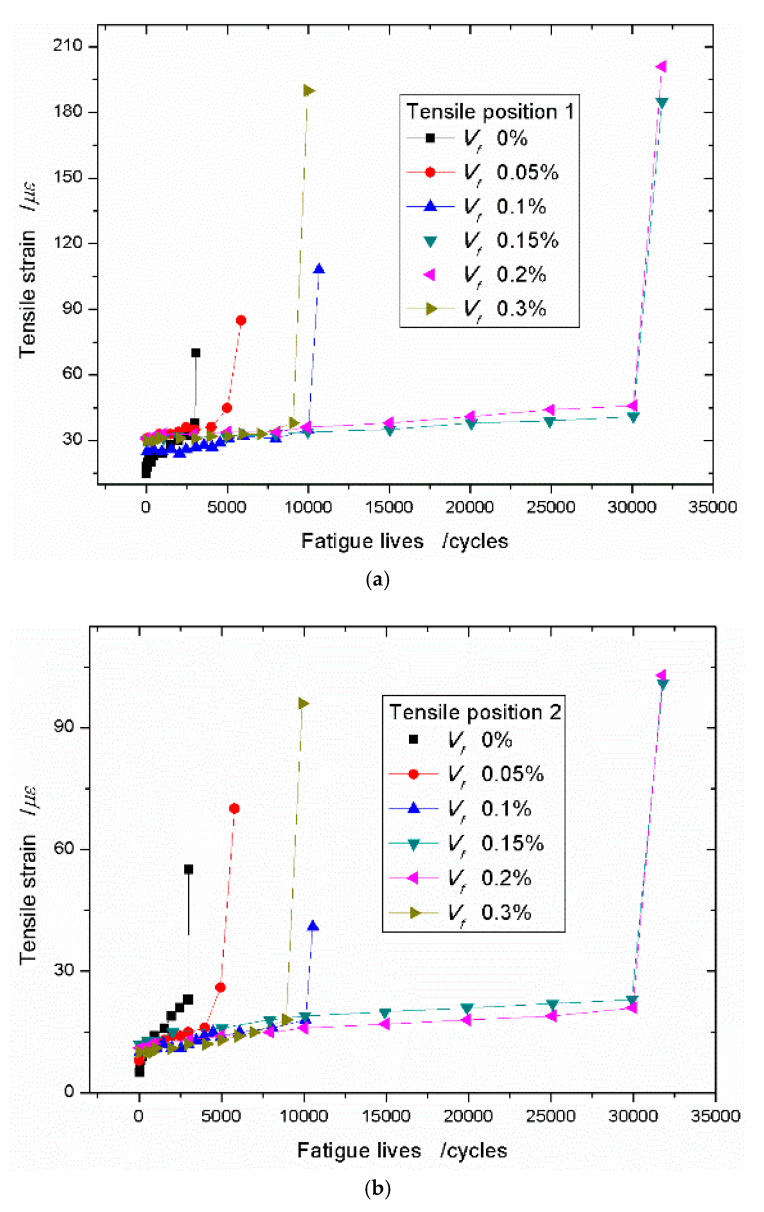
Tensile strain–fatigue life curves with different sisal fiber content under the stress level of 0.90. (**a**) Tensile position 1; (**b**) Tensile position 2.

When the stress level reaches 0.80, the improvement effects of sisal fibers on the tensile strain and the fatigue life of the composites are more obvious. For example, at position 1, the ultimate tensile strain with the sisal fiber content of 0.15% is 182 *με*, 2.3 times the value 79 *με* of the neat foamed concrete. Correspondingly, the fatigue life of the composite is 2.98 times that of the neat foamed concrete (Figure 10a). When the stress level reaches 0.90, the increments of the tensile strain and the fatigue life of the composite are 2.64 times and 10.38 times that of the neat foamed concrete (Figure 11a). Figure 10b and Figure 11b show similar results, however, comparing Figure 10a and Figure 11a, the increasing magnitudes are relatively weak.

#### 4.2.2. Fatigue Curves

(1) *S–N* Curves

**Figure 12:**
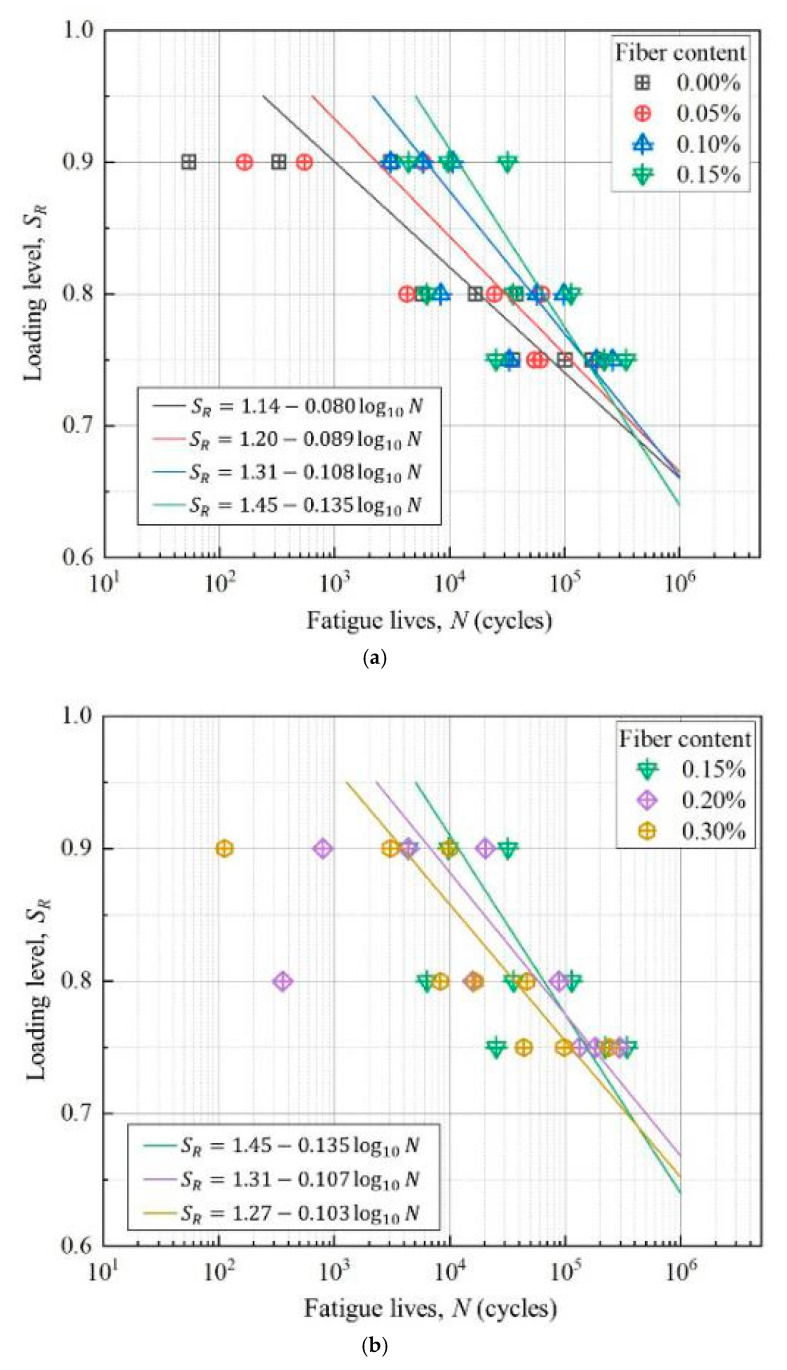
*S*_R_–*N* curves of the sisal fiber reinforced foamed concrete. (**a**) Sisal fiber content is less than 0.15% and (**b**) sisal fiber content is greater than 0.15%.

According to the fatigue experimental data shown in Figure 5, the experimental data of sisal fiber content below 0.15% and above 0.15% are grouped to obtain the *S*_R_–*N* curves shown in Figure 12a,b. As shown in Figure 12, due to the high stress/loading levels (*S*_R_ = 0.75, 0.80, 0.90), the fatigue lives of all specimens are lower. Although the experimental data are limited, the trend of *S*_R_–*N* curves is still obvious. In general, with the stress level decreasing, the fatigue life of the composite will increase. Within the scope of the experiments in this study, when the content of sisal fiber is less than 0.15%, the fatigue lives of the composite increase with the increase of fiber content, as shown in Figure 12a. When the content of sisal fiber is greater than 0.15%, the fatigue lives of the composites decrease with the increase of fiber content, as shown in Figure 12b.

For the fatigue failure of materials, because of the huge discreteness, many experiential methods are applied to analyses, such as *S–N* curve, Basquin equation, power function Equation, exponential function Equation, etc. In this study, with the limit experimental data, the following empirical Equation is used to fit the *S–N* curves.

(2) $$S_{R}=A+B\;\log_{10}\;N$$

where, *A* and *B* are constant.

By using Equation (2), the least square method is used to fit the experimental data for each *S*_R_*–N* curve, the constants A and B of each equation can be obtained, as shown in Table 6 and Figure 12. As shown in Table 6, Equation (2) can well describe the fatigue performance of sisal fiber reinforced foam concrete.

(2) Effect of Sisal Fiber Content

**Figure 13:**
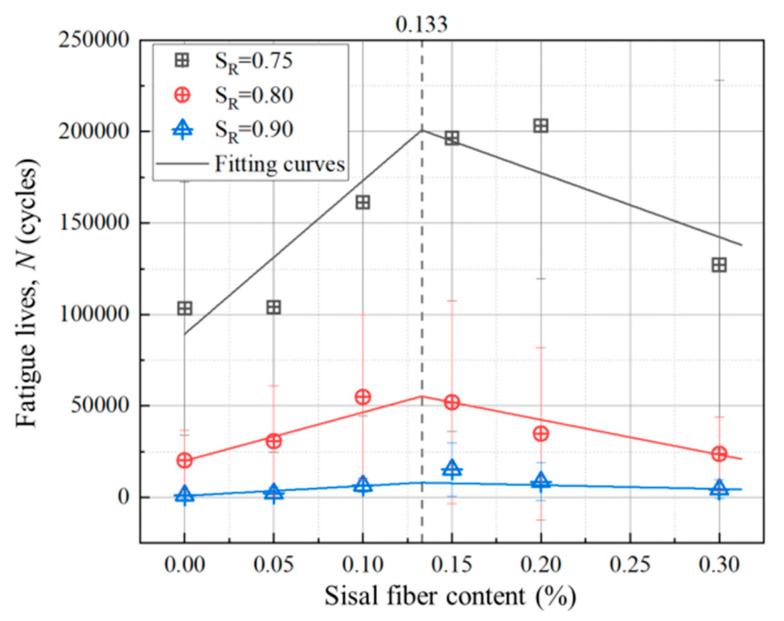
Relationships between fatigue lives and sisal fiber contents under different stress levels.

In order to discuss the effect of sisal fiber content on the fatigue performance of the composite, the average fatigue life data of various fiber content are listed in Figure 13 according to different stress/loading levels. As shown in Figure 13, for each stress/loading level, there exists the bilinear relationship between fatigue lives and sisal fiber contents, as the bilinear relationship between bending strength and sisal fiber content (Figure 3).

For each stress/loading level, the least square method is used to fit the experimental data, the bilinear equations for the relationships between fatigue life, *N* (cycles), and sisal fiber content, *C* (%), are obtained as follows:

(3) $$N=\{\begin{array}{cc} N_{0}\;+\;pC, & C\leq C_{C}\\ N_{C}-q\left(C-C_{C}\right), & C>C_{C} \end{array}$$

where, *N*_0_ (cycles) and *N*_C_ (cycles) are the fatigue lives of the concrete with no sisal fiber and that with sisal fiber content by *C*_C_ = 0.133%, respectively, and *p* and *q* are constant.

The Parameters *p*, *q*, *N*_0_, and *N*_C_ can be obtained by fitting the data of the average fatigue lives and sisal fiber contents for each stress/loading level *S*_R_, and as shown in Table 7. The fitting curves are shown in Figure 13. Table 7 and Figure 13 show that the Equation (3) can well describe the relationship between fatigue lives and sisal contents for each stress/loading level.

## 5. Conclusions

To enhance the low strength and the low toughness of the foamed concrete, sisal fibers with five contents of 0.05%, 0.10%, 0.15%, 0.20%, and 0.30% have been added into the matrix to strengthen the mechanical properties of the foamed concrete. With the static bending tests and fatigue tests, the experimental data show that the sisal fiber can improve the mechanical properties of the foamed concrete if the sisal fibers are homogeneously dispersed in the matrix. The main results are as follows:

(1) The experimental results of sisal fiber reinforced foam concrete under static bending load show that there is a double linear relationship between the content of sisal fiber and the flexural strength of the concrete. The optimal content of sisal fiber in the foamed concrete in this study can be inferred to be 0.133%.

(2) Fatigue test results show that sisal fiber can increase the fatigue life of the foamed concrete. When the content of sisal fiber is less than 0.15%, the higher the sisal fiber content, the larger the strength of the concrete. If the content of sisal fiber is greater than 0.15%, the fatigue life of the foamed concrete will decrease with the sisal fiber content increasing.

(3) For each stress level, 0.75, 0.8 or 0.9, the relationship between the fatigue life of foam concrete and sisal fiber content is efficiently analyzed by bi-linear regression. Furthermore, the bi-linear function is the same as that used to depict the relationship between the static flexural strength and the sisal fiber content. Thus, to improve the fatigue life of foam concrete, the optimal sisal fiber volume fraction is also 0.133%.

## Figures and Tables

**Table 1:** Test grouping and numbering.

Group	Sisal Fiber Content (%)	Specimen No. with Static Load Tests	Fatigue Specimen No. under Different Stress Levels		
			0.75	0.80	0.90
A0	0	0-1, 0-2, 0-3	F0-1, F0-2, F0-3	F0-4, F0-5, F0-6	F0-7, F0-8, F0-9
A1	0.05	1-1, 1-2, 1-3	F1-1, F1-2, F1-3	F1-4, F1-5, F1-6	F1-7, F1-8, F1-9
A2	0.10	2-1, 2-2, 2-3	F2-1, F2-2, F2-3	F2-4, F2-5, F2-6	F2-7, F2-8, F2-9
A3	0.15	3-1, 3-2, 3-3	F3-1, F3-2, F3-3	F3-4, F3-5, F3-6	F3-7, F3-8, F3-9
A4	0.20	4-1, 4-2, 4-3	F4-1, F4-2, F4-3	F4-4, F4-5, F4-6	F4-7, F4-8, F4-9
A5	0.30	5-1, 5-2, 5-3	F5-1, F5-2, F5-3	F5-4, F5-5, F5-6	F5-7, F5-8, F5-9

**Table 2:** Mix ratio of the neat foamed concrete.

Cement (kg)	Sand (kg)	Fly Ash (kg)	Silica Fume (kg)	Water (kg)	Water Reducing Agent (kg)	Sisal Fiber Content (%)
588	360	168	84	378	3	0

**Table 3:** Mechanical properties of sisal fiber.

Length (cm)	Purity (%)	Moisture Regain (%)	Fiber Tension (N)	Elongation (%)	Density (gcm^−3^)
90–130	>97	9.5	>800	5	1.34

**Table 4:** Results of the bending tests.

No.	Average Max. Load (kN)	Bending Strength (MPa)	Increasing (%)	S-Ultimate Strain (με)	M-Ultimate Strain (με)
0	2.76	0.83	0	41	93
1	3.13	0.94	13	78	123
2	3.31	1.00	20	106	175
3	3.58	1.07	29	131	582
4	3.17	0.95	14	120	368
5	2.95	0.89	7	101	231

Note: S—support point; M—mid-span.

**Table 5:** Load levels with different sisal fiber contents.

Stress/Loading Level *S*_R_	Sisal Fiber Content											
	0%		0.05%		0.1%		0.15%		0.2%		0.3%	
	P_max_	P_min_	P_max_	P_min_	P_max_	P_min_	P_max_	P_min_	P_max_	P_min_	P_max_	P_min_
0.75	2.07	1.04	2.35	1.18	2.48	1.24	2.68	1.34	2.43	1.22	2.21	1.11
0.80	2.21	1.11	2.50	1.25	2.65	1.33	2.86	1.43	2.60	1.30	2.36	1.18
0.90	2.48	1.24	2.82	1.41	2.98	1.49	3.22	1.61	2.92	1.46	2.66	1.33

**Table 6:** Parameters A and B.

Content of Sisal Fiber	A	B	R^2^ for Equation (2)
0%	1.14	−0.080	0.999
0.05%	1.20	−0.089	0.999
0.10%	1.31	−0.108	1.000
0.15%	1.45	−0.135	0.955
0.20%	1.31	−0.107	0.937
0.30%	1.27	−0.103	0.965

**Table 7:** Parameters of the Equation (3).

Stress Level, *S*_R_	*N*_0_	*N*_C_	*p*	*q*	R^2^ for Equation (3)
0.75	89,354	200,808	8.38 × 10^7^	3.50 × 10^7^	0.766
0.80	29,914	55,259	2.65 × 10^7^	1.91 × 10^7^	0.918
0.90	917	8099	5.40 × 10^6^	2.07 × 10^6^	0.826
